# Supplementary material for: MetaRibo-Seq measures translation in microbiomes
Source: Nat Commun. 2020 Jun 29;11:3268. doi: 10.1038/s41467-020-17081-z (PMC7324362; doi:10.1038/s41467-020-17081-z)
Supplement: Supplementary file 10 — Supplementary Data 7 [file 41467_2020_17081_MOESM10_ESM.zip › File2/Confidence_High_Taxonomy/57547_out.krona.html]

Javascript must be enabled to view this page.

members
magnitude
magnitudeUnassigned
count
unassigned
taxon
rank

57547\_out

2

superkingdom
2
2

phylum
1
1239

186801
1
class

1
186802
order

family
1
186806

genus
1730
1

1
39485

SRS011134\_contig\_number\_46656
species

976
1
phylum

200643
1
class

171549
1
order

171552
1
family

species

SRS022609\_contig\_number\_38256
1
2049047
